# Ensuring the Safety of Sunscreens, and Their Efficacy in Preventing Skin Cancers: Challenges and Controversies for Clinicians, Formulators, and Regulators

**DOI:** 10.3389/fmed.2019.00195

**Published:** 2019-09-04

**Authors:** Sharad P. Paul

**Affiliations:** ^1^Faculty of Design and Creative Technologies, Auckland University of Technology, Auckland, New Zealand; ^2^School of Medicine, University of Queensland, Brisbane, QLD, Australia

**Keywords:** skin, skin cancer, sun protection, sunscreens, melanoma, basal cell cancer, squamous cell cancer

## Abstract

When people think about sun-protection or prevention of skin cancer, sunscreens readily come to mind. Sunscreen effectiveness is tested *in vivo* by the ability to prevent erythema of skin, yet testing methods vary between markets, and many sunscreens fail to achieve their claims. This article discusses the mechanism of action of sunscreens, Sun Protection Factor (SPF), safety concerns and the challenges for regulators. Many sunscreens that prevent erythema do not provide adequate protection as they contain anti-inflammatory agents; others have ingredients whose risks have not been fully evaluated. This article reviews the imperfect science behind sunscreens and points out the gaps in knowledge regarding safety, efficacy, public knowledge, and perception. Regulations vary between countries and only adds to the confusion. To truly prevent skin cancer, clinicians, formulators and regulators need to come together to research more and improve public education.

## Introduction

Over a decade ago, an article discussing sunscreens and SPF (Sun-Protection Factor) noted that “sunscreen with an SPF of 30 may provide an effective SPF-rating of only 2 against UV- A” (1). Until then UV-B rays (280–320 nm) were considered the main cause of skin cancer—for example, the FDA Panel on sunscreens noted “the lower wavelength limit of cancer-producing radiation on the skin of mice and rats has been shown to be 325 nm, i.e., the same spectral range that produces sunburn in human skin” (2).

The same year, it was noted the basal layer of skin in cutaneous squamous cell cancers (cSCC) harbored more UVA than UVB fingerprint mutations, indicating a role for UVA in human skin carcinogenesis (3). The basal location of UV-A (rather than UV-B) mutations indicated that longer wavelength (320–400 nm) UV-A rays may be a bigger culprit in the pathogenesis of skin cancer. However, others have noted using immunohistochemical analysis that the NOTCH1 gene correlates with mutation status in sporadic cSCC, and regions of NOTCH1 loss or down-regulation are frequently observed in normal looking skin—meaning that NOTCH1 may be a gatekeeper in human cSCC (4). It was also suggested that cutaneous SCC harbors a greater burden of mutations when compared with common malignant tumor types (4).

In 2001, the International Agency for Research on Cancer reviewed 15 case-control studies examining sunscreen use and melanoma and concluded there was “insufficient evidence” that sunscreen formulations protected against melanoma and basal cell carcinoma (BCC) and “limited evidence” for protection against cutaneous squamous cell carcinoma (cSCC) (5). One of the problems in studying the epidemiology of sunscreens is that people using sunscreen also have more sun-exposure and therefore a higher risk of skin cancer, leading to difficulties designing a randomized-controlled trial (RCT). Only one RCT at Nambour in Australia has shown “daily sunscreen use” halved the number of melanomas and reduced the number of SCC, but not BCC (6). This must also be viewed in the context that the Nambour trial was that of a white population in a condition of extreme ambient UV. Further, it has been hypothesized that participants in the Nambour Trial were too old to prevent the onset of BCC (7) and therefore the findings may not be reproducible in studies of other population groups or geographical locations. Given the incidence of melanoma is increasing faster than that of any other solid tumor (8) and its associated mortality, it is time to review the uses of sunscreen in prophylaxis, adequacy of sunscreen formulations and the role of regulators in ensuring that the public is aware and also adequately protected.

## Do Sunscreens Really Protect Against Skin Cancer?

Sun Protection Factor (SPF) is a measurement that is used in the ratings of sunscreens. Simplistically it has been explained in popular literature that (when properly applied) SPF 15 lets in one in 15 harmful UV rays (93% protection), while SPF 30 lets in one in 30 (97% protection), and SPF 50 lets in one in 50 UV rays (98% protection) in an effort to educate the public that differences between SPF 30 and 50 are minimal (9). Scientifically, sun protection factor is defined as the ratio of the least amount of ultraviolet energy required to produce a minimal erythema on skin protected by sunscreen to the amount of energy required to produce the same erythema on unprotected skin i.e., the minimal erythema dose (10).

It has been known that UV-radiation damage is directly absorbed by DNA and leads to the formation of pyrimidine dimers. This DNA damage is repaired by groups of enzymes that “excise” these dimers and replace them with the correct sequences. When this repair mechanism fails, permanent mutations can occur (11). Sunscreen effectiveness is tested *in vivo* by the ability to prevent erythema of skin and we know that around 300 nm is the typical action spectrum that induces both erythema and the formation of pyrimidine dimers (11).

Genomic inheritance analysis facilitates the identification of alleles that cause genetic disorders and studies the mutations that cause them (12). Given that there are around 10^12^ stem cell divisions per day in the adult human body (13), if cancer was caused by a single-cell mutation, then the theoretical risk of cancer would be 10^12^ stem cell divisions × 1.1 × 10^−8^ point-mutations [because the point mutation rate is mutation rate is 1.1 × 10^−8^ per cell division (13)], meaning everyone would get cancer daily. This is obviously not the case, and this led to the multiple-hit concept of oncogenesis for melanoma caused by UV-damage to skin. This theory particularly fits the human melanoma models for several reasons and explains tumor progression, as well as steep climb in incidence of tumors with age:

1. The total number of naevi, either acquired or that a typical a person has is a very good predictor of that individual's risk of developing melanoma (14)
2. Multiple blistering sunburns during childhood are associated with an increased number of naevi and also melanomas (15)
3. Indeed, sunburns at any age do increase melanoma risk with a meta-analysis showing that the magnitude of risk for 5 sunburns per decade is highest for adult and lifetime sunburns (16)
4. Melanoma inhibitory activity (MIA) is expressed in both naevi and melanomas but not in normal melanocytes (17); further this MIA is p53 dependent and UV-light induces MIA activity (18)

It is now established that UV-induced skin damage is responsible for cutaneous melanoma. Further, there is a high frequency of BRAF gene-mutations in cutaneous melanomas as compared with uveal or mucosal melanomas, suggesting a link between BRAF mutations and UV exposure (19). Whole-exome and whole-genome sequencing of melanomas have shown many UV signatures in melanomas from sun-exposed body sites suggesting that some BRAF mutations can be caused by “non-informative UV-induced mutations” i.e., changes that do not occur at a dipyrimidine sites (20, 21).

Mutations of the p53 gene are different in sun-damaged skin and other organs—other tumors have different mutations—A to T and G to T transversions, rather than C to T or CC to TT p53 mutations that occur in skin (22) and therefore some groups have studied the effect on these p53 mutations as a way of evaluating sunscreen efficacy. As noted by a review from Australia, sunscreens do reduce these p53 mutations and therefore sunscreens in general can be expected to reduce the risk of skin cancer (23).

## Concerns Regarding the Safety of Sunscreens

### Hormonal Effects of Chemicals in Sunscreens

There is increasing public concern regarding the harmful effects of chemicals in sunscreens and the science is worth reviewing. The three main classes of chemicals that are the main cause of concern are benzophenones, camphor derivatives (such as 4-methyl benzylidene camphor or 3-benzylidene camphor) and cinnamate derivatives (such as octyl methoxycinnamate, isopentyl-4-methoxycinnamate; octocrylene).

Benzophenone used to be widely used in sunscreen products, but residues of benzophenone were noted not just in wastewater, but also in human urine and breast milk (24, 25), and many studies have shown both estrogen and androgen disrupting effects—for example, estrogen disrupting effects seen in animal studies include inhibition of the activity of 17β-oestradiol and the proliferation of MCF-7 cell lines, while benzophenone-like UV filters caused marked developmental and reproductive toxicity in fish and rat studies (26). Benzophenones disturbed normal hormonal levels of testosterone during male development of mice and rats by inhibiting the conversion of androstenedione to testosterone, and this decreased androgenic activity persisted even after metabolism mediated by rat and human liver microsomes (27). Benzophenones also affect the thyroid-hypothalamus axis by inhibiting or inactivating the activity of thyroid peroxidase disturbing the biosynthesis of thyroid hormone (28).

Camphor derivatives are not used as filters in sunscreens but as UV B-absorbers. These accumulate in tissues after prolonged exposure, and being very lipophilic, they can be easily absorbed after direct contact with the skin (29). 4-methyl benzylidene camphor or 3- benzylidene camphor have shown anti-oestrogenic or pro-progesterone activity in various fish, mammals and cell-based bioassays (30).

Cinnamate derivatives such as octyl methoxycinnamate and octocrylene are widely used in current sunscreens as they are capable of absorbing both UV-A and UV-B, especially in the 305 nm range. Octyl methoxycinnamate is approved for use in sunscreens in both the USA and EU, however multiple studies have shown these chemicals can disrupt many different hormones including estrogen, progesterone and thyroid hormones (31).

Given the application of sunscreen is not dose-controlled, it is difficult to design a proper RCT to study toxicity, and therefore we have to rely on animal-studies to provide a guide. One cannot dismiss customer safety concerns regarding sunscreens without continuing to study them and more research is needed. In fact, in the US State of California, sunscreens must carry this warning on labels of products containing the cancer-causing chemical benzophenone: “WARNING: This product contains benzophenone, a chemical known to the State of California to cause cancer” (32).

### Anti-inflammatory Chemicals in Sunscreens and the Masking of Sunburns

In March 2013, Dr. Robert Sayre, a sunscreen researcher submitted a “citizen petition” to the Food and Drug Administration (FDA) requesting the Commissioner of Food and Drugs to amend approval for certain sunscreen agents. His petition specifically requested the FDA formally withdraw approvals of the anti-inflammatory sunscreen ingredients such as dioxybenzone, oxybenzone, trolamine salicylate, homosalate, and octisalate (33). Interestingly, these agents especially homosalate and octisalate are widely used in sunscreens today. However, the anti-inflammatory actions of these ingredients mean these agents suppress UV-induced erythema (which is how sunscreens are tested *in vivo*) by means other than attenuation of radiation i.e., these agents may mask sunburn without preventing cellular/genomic damage which is the rationale for using these in the first place. The FDA had previously dismissed this possibility using the argument that it was unlikely anti-inflammatory ingredients would affect SPF values because suppression of erythema is relatively short-lived—compared to the 16–24 h interval between UV exposure and erythema observed in an SPF test subject (34). However, Sayre and collaborators reported that “clearly the sunscreen product altered not only both the early and the delayed erythemic responses but also longer-term pigmentation responses” (there was a rebuttal from the Editor of the journal in which this article appeared) (35).

Couteau found bisabolol derived from chamomile or glycyrrhizate from liquorice had potent anti-inflammatory effects on skin in laboratory studies persisting for several hours (36). The study was unable to quantity the relationship between the SPF value and anti-inflammatory activity of sunscreens (36). However, more recently a study demonstrated a contrary view that commonly-used anti-inflammatory or anti-oxidants at concentrations typically used in sunscreen products, neither influenced SPF value nor delay erythemal response, concluding SPF values are good indicators of photoprotective capacity (37).

As explained earlier, there has been only one RCT that has shown sunscreens prevent skin cancer. If anti-inflammatory agents reduce redness and thereby mask erythema, users and physicians recommending such products may have a false sense of security. Cis-urocanic acid, formed by the photoisomerization of trans-urocanic acid is now known to be a mediator of the immunomodulation that is caused by UV exposure and studies have shown production of cis-urocanic acid is reduced significantly (*p* < 0.01) when sunscreens are applied in an amount lower than recommended (38). Noonan and others hypothesize the photoreceptor for systemic UV-induced immunosuppression of contact hypersensitivity may be urocanic acid and the same agent plays a role in UV-induced carcinogenesis via the production of tumor-specific suppressor cells (39).

### Anti-oxidants and Vitamin A Derivatives

Many sunscreens use botanicals capable of anti-oxidant activity to, in theory at least, mop up some free radicals caused by sun damage. Some studies reported the addition of antioxidant vitamins and botanicals like caffeine or echinacea to formulations have reduced sun damage (40). Vitamin A derivatives such as retinols are common in “anti-aging” cosmetics and sometimes used in sunscreens. Some research shows such vitamin A additives may actually speed the development of tumors and lesions on sun-exposed skin as UV rays can break down antioxidants, forming harmful by-products (41).

### Technical Issues With Some UVA Filters

There has been criticism the US FDA only allows zinc oxide and titanium dioxide as UVA filters. The aforementioned are *physical* filters used in mineral sunscreens. However, a *chemical* filter such as avobenzone is often recommended and this is the primary UVA filter in non-mineral sunscreens (outside the USA). Avobenzone degrades when exposed to UV rays and therefore manufacturers are forced to improve stability by mixing avobenzone with other active ingredients such as octocrylene which is also not free of safety concerns (42).

Some of the concerns described above, such as the endocrine effects have been demonstrated mainly in animal studies. However, public concerns regarding the safety are real and hence it is important to evaluate these concerns with further clinical studies.

## Regulatory Aspects of Sunscreens

If sunscreens are to be therapeutic or prophylactic against skin cancer, then regulations are more about public health than about testing requirements. For example, tanning salons have been banned in many jurisdictions. However, British researchers such as Diffey et al. estimated that if someone with fair skin was using a poor-quality sunscreen, a 2-week vacation to the tropics could provide as much UVA exposure as he or she would get by visiting a tanning salon 10 times! (43).

The real measure of the clinical effectiveness of a sunscreen is its ability to reduce biological effects like DNA damage, immune system suppression and free radical generation—all of which are precursors to skin cancer. But these biological studies are not often done as they are both expensive and difficult to do. So how are sunscreens tested?

### *In vivo* Testing

In principle, the evaluation of sunscreen efficacy on human subjects seeks to measure the response of the human body to sunscreen application. But all over the world, irrespective of jurisdictions, sunscreens do not seem to measure up to the claims.

In the USA, sunscreens are considered “over the counter” (OTC) drugs and are considered under the FDA's Final Rule 2011 and the Sunscreens Innovation Act 2014 (44). The FDA demands sunscreens are tested in 10 human volunteers under a high-intensity UV lamp and reddening of skin is evaluated the next day. The FDA allows manufacturers to discard three out of 10 test subjects and the SPF value on the label is the amount of UV that caused a sunburn in the remaining seven subjects (45). All jurisdictions essentially follow the same principles of *in vivo* testing with minor variations.

In the US, the FDA has capped SPF values at 50+, calling ultra-high SPF values “inherently misleading” as they can provide consumers with a false sense of security (46). During *in vivo* testing, a thick layer of sunscreen is applied. In this regard, one must understand that sunscreen protection is not related proportionally to the thickness of product applied to human skin. Consumers may sometimes achieve an even lower than expected sunburn protection from high SPF products than from low SPF sunscreens (47).

Some people consider such *in vivo* testing requirements border on “unethical” (48) because they expose volunteers to potentially dangerous UV exposure and some suggest adopting a different method for evaluating UVA protection in humans by using the immediate pigment darkening (IPD) test which exposes volunteers to shorter UVAII rays and examines the amount of skin tanning that results. While photobiologists reckon that the results should be similar between these different modalities, the IPD value does not reflect the amount of protection a sunscreen provides from lower-energy UVAI rays and therefore has not been adopted.

### *In vitro* Testing

While this is not done as part of mandatory requirements, *in vitro* testing avoids the variations and ethical issues of testing human volunteers. These tests do not measure the prevention of tanning but quantify UVA protection in the UVAI spectrum. These are then converted to a UV protection factor. The issue is the composition of the testing slide (quartz or acrylic) that the surface roughness of the sunscreen being tested on the slide can affect measurements, and therefore *in vitro* testing has also not been adopted.

### *In silico* Testing

*In silico* testing calculates how much light will pass through a sunscreen based on its ingredients and the expected UVB and UVA protection. It is said to avoid pitfalls of human testing and *in vitro* testing. BASF, one of the largest manufacturers of sunscreen ingredients has developed a scale that simulates sunscreen performance. The BASF sunscreen simulator has been shown to have a “very good correlation between SPF *in-silico* and SPF *in-vivo* and provides realistic estimations of the final product performance” (49).

However, currently most countries use *in vivo* testing in human subjects as the basis for testing sunscreen efficiency and therein also lies the problem with different and variable results and regulation.

## Certification of Sunscreens

The regulation of sunscreens is often seen as the best way to resolve these issues but in several areas, as we have discussed in this review, the science is lacking. Australia and New Zealand are good case studies as they have the highest UV indices in the world, but differing levels of oversight. In Australia “therapeutic” sunscreens (any cosmetic that claims SPF >4) have to be regulated by the TGA (Therapeutic Goods Administration). In New Zealand, testing is not mandatory and many organizations, including Consumer NZ, have called for testing to be mandatory (50). But what is interesting is that this does not seem to alter the fact that even in Australia where testing and TGA-listings of sunscreens are mandatory, many sunscreens do not live up to their claims. Choice, an Australian consumer watchdog tested 6 SPF 50+ sunscreens in late 2015 and found most did not live up to their SPF claims. These were from different brands and marketed to the general public, some targeted at kids and sportspeople, and were tested according to the Australian Standard (51). Similar testing of sunscreens in the USA and UK also showed the same results i.e., many sunscreens did not pass the claimed standard on testing, indicating that because testing is done on human subjects' skin, SPF results are always likely to be variable. Many sunscreens contain untested anti-inflammatory agents, which allow them to pass regulations based on the assessment of skin erythema, but it does not make them therapeutic.

Perhaps a more honest strategy is to follow the suggestions made by the European Commission, which rather than relying on SPF values, suggests simplifying sunscreens into four broad SPF categories: low sunburn-protection, moderate sunburn-protection, high sunburn-protection, and very high sunburn-protection (52). Avoiding or regulating against the use of anti-inflammatory agents and making this “sunburn-protection” grading a global standard may be a more scientific approach. To really reduce the incidence of skin cancers, public education about the imperfect science behind sunscreens and sun-protection is also important. Recently, a paper published in the JAMA looked at the plasma concentrations of the main sunscreen ingredients for the first time (53). The authors tested for levels of avobenzone, oxybenzone, octocrylene, and ecamsule (the most common active ingredients) in people after sunscreen application. All four chemicals achieved blood levels higher than that 0.5 ng/mL threshold, a standard used to determine the need for toxicity studies.

Previously, it was thought that the systemic absorption of sunscreen ingredients was minimal in chemical sunscreens (as opposed to mineral sunscreens). In fact, with the exception of ecamsule in the JAMA study, far higher concentrations were noted of the other chemicals. The average peak oxybenzone level was around 200 ng/mL—that is 400 times higher than the level to trigger a formal toxicology review. The authors concluded the systemic absorption of sunscreen active ingredients supports the need for further studies to determine the clinical significance of these findings (53) and this is something regulators such as the FDA need to address promptly to allay public (and physician) concerns.

### Educating the Public Regarding Sunscreens and Sunscreen Use

Australia, given its high UV radiation levels has an environment that means two in three Australians will be diagnosed with skin cancer before the age of 70 (54). It is recommended people venturing into the outdoors during sunshine hours should wear a hat, sunscreen with an SPF of 30 or higher, clothing that covers skin, and stay in the shade (55). In a survey done in Australia during the 1998–2004 period among adolescents, use of sunscreen was the most frequently practiced sun-protection behavior—similar to findings in US and European adolescents (56). The use of hats significantly has however decreased between 2001–2002 and 2011–2012 whereas the use of sunscreen and wearing protective clothes were unchanged, indicating the importance of ongoing public education (57). Wearing protective clothing was the least frequent behavior across all survey years (57). Another study done in Norway revealed the prevalence of sunscreen use increased from 1997 to 2007 but this increase was not accompanied by a decrease in sunburn (58) and this may reflect the use of anti-inflammatory agents discussed earlier. As many authors have stated, more effort and education is needed to encourage greater enactment of sun-protection behavior (57).

It is this author's experience that people often state unfounded concerns that sunscreen use may interfere with their body's vitamin D production as a reason for not using sunscreen products. However, a recent study that looked at sunscreen use during holidays at high UV- index locations found sunscreens allow sun-protection without interfering with vitamin D synthesis (54). Further, a high UVA-protective sunscreen actually enables better vitamin D synthesis than a low UVA sunscreen because the former transmits more UVB than the latter (59). “Good intentions, bad practices” was the title of a paper discussing sun-protection practices in early childhood in New Zealand (60). That may sum up we have been doing with sunscreens all along—with inadequate education and research, lack of toxicity studies and uniform regulations.

## Conclusion

Skin cancer is an environmental cancer—with UV radiation the major causative agent. Sunscreens have long been part of the strategy for protection against skin cancer, however there are gaps in research and knowledge regarding safety, efficacy, and also public perception. Regulations vary between countries and are not uniform, adding to confusion in a global market. To truly prevent skin cancer, clinicians, formulators, and regulators need to come together with improved research, safer formulations, and public education.

## Author Contributions

The corresponding author solely conceived and designed the analysis, collected data, performed the analysis, and wrote the paper.

### Conflict of Interest Statement

SP is the Director of the company Sharad Paul Skincare Ltd. This article was written independently fromthe author's involvement in the company.
